# School health promotion during the COVID-19 pandemic: Associations with school leaders’ health literacy: Simona Betschart

**DOI:** 10.1093/eurpub/ckac129.659

**Published:** 2022-10-25

**Authors:** S Betschart, A Sandmeier, G Skedsmo

**Affiliations:** Schwyz University of Teacher Education, Goldau, Switzerland

## Abstract

**Background:**

School leaders are credited with an important role when it comes to school health promotion. During the COVID-19 pandemic, much health-related information was available and had to be interpreted and acted upon by school leaders. Therefore, it is crucial that they have sufficient health literacy as the ability to gain access to, understand and use health-related information. A study in Germany showed that limited health literacy among school principals was associated with low levels of health promotion activities. This paper explores the association between school leaders’ health literacy and school health promotion in Switzerland, addressing the following questions: 1. What is the relationship between health literacy of school leaders and the implementation of school health promotion? 2. Does health literacy explain variance above and beyond other antecedents, such as principals individual mental health and attitudes?

**Methods:**

The quantitative analysis is based on an online survey conducted among school leaders in the German- and French speaking parts of Switzerland in June 2021 as part of the project “COVID-19 health literacy school principals survey”. The final sample comprised N = 339 school leaders. The data were analyzed using stepwise regression with health literacy, stress, wellbeing and attitudes toward school health promotion as antecedents and COVID-19 related school health promotion as the outcome.

**Results:**

The results show that health literacy of school principals has played an important role in the implementation of school health promotion during the COVID-19 pandemic. It explains additional variance beyond other antecedents.

**Conclusions:**

One way to promote implementation of school health promotion is to strengthen the health literacy of school leaders.

